# Lung Large Cell Neuroendocrine Carcinoma: A Population-Based Retrospective Cohort Study

**DOI:** 10.3390/jcm12124126

**Published:** 2023-06-19

**Authors:** Xiaoli Mu, Dan Pu, Yajuan Zhu, Yixin Zhou, Qiang Wu, Qing Liu, Liyuan Yin, Yan Li

**Affiliations:** 1 The Department of Biotherapy, Cancer Center, West China Hospital, Sichuan University, Chengdu 610041, China; 2 Lung Cancer Center, West China Hospital, Sichuan University, Chengdu 610041, China; 3 Department of Radiation Oncology, Cancer Center, West China Hospital, Sichuan University, Chengdu 610041, China

**Keywords:** pulmonary large cell neuroendocrine carcinoma, disease-specific survival, prophylactic cranial irradiation, nomogram

## Abstract

Backgrounds: Pulmonary large cell neuroendocrine carcinoma (LCNEC) is a rarely high-grade neuroendocrine carcinoma of the lung with features of both small cell and non-small cell lung cancer. In this study, we aim to construct a prognostic nomogram that integrates the clinical features and treatment options to predict disease-specific survival (DSS). Methods: A total of 713 patients diagnosed with LCNEC were from the US National Cancer Institute’s Surveillance Epidemiology and End Results (SEER) registry between 2010–2016. Cox proportional hazards analysis was conducted to choose the significant predictors of DSS. External validation was performed using 77 patients with LCNEC in the West China Hospital Sichuan University between 2010–2018. The predictive accuracy and discriminative capability were estimated by the concordance index (C-index), calibration curve, and receiver operating characteristic (ROC) curve. The clinical applicability of the nomogram was verified through the decision curve analysis (DCA). Additionally, we conducted a subgroup analysis of data available in the external cohort that may impact prognosis but was not recorded in the SEER database. Results: Six independent risk factors for DSS were identified and integrated into the nomogram. The nomogram achieved good C- indexes of 0.803 and 0.767 in the training and validation group, respectively. Moreover, the calibration curves for the probability of survival showed good agreement between prediction by nomogram and actual observation in 1-, 3- and 5-year DSS. The ROC curves demonstrated the prediction accuracy of the established nomogram (all Area Under Curve (AUC) > 0.8). DCA exhibited the favorable clinical applicability of the nomogram in the prediction of LCNEC survival. A risk classification system was built which could perfectly classify LCNEC patients into high-, medium- and low-risk groups (*p* < 0.001). The survival analysis conducted on the West China Hospital cohort indicated that whole brain radiation therapy (WBRT), prophylactic cranial irradiation (PCI), surgical procedures, tumor grade, Ki-67, and PD-L1 expression were not significantly associated with DSS. Conclusion: This study has effectively developed a prognostic nomogram and a corresponding risk stratification system, which demonstrate promising potential for predicting the DSS of patients with LCNEC.

## 1. Introduction

Pulmonary large cell neuroendocrine carcinoma (LCNEC) is a rare but highly aggressive pathological type of lung cancer [1,2], which was first reported by Travis et al. in 1991 [3]. According to a retrospective study conducted by Shah et al. LCNEC accounted for approximately 3% of all cases of pulmonary neuroendocrine carcinoma [4]. The majority of patients diagnosed with LCNEC were middle-aged men with a significant history of smoking [5]. The pathological manifestations of LCNEC are complex, and preoperative small biopsies are difficult to distinguish it from some poorly differentiated cancers. It is reported that 25–27% of biopsy specimens of LCNEC are misdiagnosed as non-small cell lung cancer (NSCLC) or small cell lung cancer (SCLC) [6]. Therefore, surgical resection of specimens is often required to achieve pathological diagnosis [7]. Owing to the rarity of LCNEC and the consequent paucity of available data, there is a dearth of prospective, randomized trials to establish the optimal therapeutic approach for this malignancy. As such, the optimal treatment strategy for LCNEC remains elusive. Current guidelines recommend surgical resection as the primary treatment for all non-metastatic stages of LCNEC, which is the same as the treatment of NSCLC [8,9]. However, similar to SCLC, LCNEC exhibits a high degree of biological invasiveness, which makes it prone to recurrence and metastasis [9,10]. Therefore, even in its earliest stages, surgery alone may be insufficient to treat LCNEC. A Spanish multicenter study found that the 5-year survival rate of LCNEC was 21% [11]. Moreover, other studies found that even for stage I LCNEC, the 5-year survival rate was only 27~67%, which is significantly lower than that of other types of stage I NSCLC [6,12,13]. Due to the relative scarcity of cases and difficult diagnosis, the prognostic characteristics of LCNEC patients have not been widely explored.

More and more studies used nomograms to construct prediction models to clarify the risk of individual patients and guide the clinical decision-making of patients through intuitive images [14,15]. Recently, Ma et al. [16] have developed a nomogram to predict the DSS for LCNEC based on the Surveillance, Epidemiology, and End Results (SEER) database; however, it lacked independent external data to verify the model, and the ability of the model based on foreign population to predict the risk of LCNEC patients in China is not clear. As a result, the current study built and validated a novel predictive model for predicting the prognosis of LCNEC patients utilizing a cohort from the SEER database and an external validation cohort of Chinese patients with LCNEC. Additionally, we performed a subgroup analysis of the external cohort to obtain a more comprehensive understanding of the prognostic factors associated with LCNEC.

## 2. Material and Methods

### 2.1. Patients

Relevant information on patients diagnosed with LCNEC from 2010 to 2016 was collected through the SEER program of the National Cancer Institute (http://seer.cancer.gov/, accessed on 1 February 2022). SEER database is a unique research resource for oncology practice in the United States (US). It collects tumor diagnosis, treatment, and survival data for about 30% of the US population. The inclusion criteria for this study were patients diagnosed with LCNEC (ICD-O-3 Code: 8013/3) between 2010 and 2016, the extracted clinical information included age, gender, grade, race, laterality, T classification, lymph node status, distant metastasis, brain metastasis, clinical stage, treatment (including surgery, radiotherapy, and chemotherapy), surgical procedures, cause-specific death classification, survival status, and survival time. Exclusion criteria were as follows: (a) patients whose clinical data is inadequate or missing and (b) patients who died of causes other than LCNEC. According to the above standards, a total of 713 eligible cases were selected as the training group.

At the same time, 77 patients in West China Hospital of Sichuan University from 2010 to 2018 were selected as the external validation cohort, and the corresponding clinical information was collected from the electronic medical records. The inclusion and exclusion criteria were the same as above, and we collected additional information, including pathological differentiation, grade, and Ki-67 percentage, and PD-L1 expression, to provide a more comprehensive analysis of the clinical and pathological characteristics of the LCNEC patients included in our study. This study was approved by the Institutional Review Board of West China Hospital of Sichuan University.

### 2.2. Endpoint Definition

The endpoint of the current study was disease-specific survival (DSS), which was the interval between the initial diagnosis of LCNEC and the occurrence of LCNEC-specific death.

### 2.3. Statistical Methods

All data were statistically analyzed using the IBM SPSS 22.0 program (IBM Corporation, Armonk, NY, USA), and R 4.0.2 software (http://www.r-project.org, accessed on 1 October 2020) was used to construct and validate the nomogram. The effects of various factors on DSS were calculated by the Kaplan–Meier method, and the survival differences between the groups were compared by log-rank test. Univariate Cox regression was used to screen potential prognostic factors in the training cohort, the variables with *p* < 0.1 in univariate analysis were included in multivariate Cox regression analysis, and the nomogram model was constructed based on the multivariate Cox regression model.

The concordance index (C-index), receiver operating characteristic (ROC) curve, and calibration curve (1000 bootstrap resamples) were used to evaluate the prediction ability and accuracy. The higher the C-index is, the better the prediction of the model might be. The calibration curve was used to compare the actual and predicted results, with a perfect prediction corresponding to a slope of 1 (represented by the gray line at a 45-degree diagonal). The area under curve (AUC) value of the ROC curve represents the discrimination of the model, whereby a larger AUC indicates a higher diagnostic accuracy. The decision curve analysis (DCA) is a valuable tool that is utilized to explore the impact of a model on the clinical net benefit rate across various positive thresholds. The significance of DCA lies in its ability to provide a more comprehensive and clinically relevant evaluation of a model’s performance. In our research, we employed the DCA to weigh the risks and benefits at different times. Subsequently, we established a risk stratification system based on the risk scores obtained by predicting patients according to the nomogram. The optimal cut-off value for the risk scores was calculated using X-Tiles software (Version 3.6.1), and patients were classified into low-, medium-, and high-risk groups accordingly. Due to the limitations of the SEER database, some clinical factors that affect prognosis were not recorded. Therefore, we conducted a subgroup analysis of the available data from the external cohort to comprehensively identify possible prognostic factors. All statistical tests were two-sided.

## 3. Results

### 3.1. Patients Characteristics

In the training set, the SEER database yielded a total of 713 eligible patients from 2010 to 2016, with a median follow-up time of 45 months. The majority of cases were male (54.7%) and middle-aged. Most tumors had the right laterality (57.6%) and a poor degree of differentiation (73.2%). Distribution of the TMN stage was 27.1%, 14.0%, 19.1%, and 39.8% for I, II, III, and IV, respectively. While more than half (52.0%) of patients had lymph node metastasis, 39.8% of patients with distant metastasis, and among them, 13.9% with brain metastasis. A total of 48.0% of patients received surgery, with 95% of those undergoing lobectomy, a few patients (14.3%) received radiotherapy, and 55.5% of patients were treated with chemotherapy.

In terms of the validation cohort, 77 LCNEC patients in West China Hospital were finally included. The median follow-up time was 37 months and the median age of patients in the validation set was 61 years old. The majority of patients (66.2%) received surgery, and among those who underwent surgery, 90.2% had a lobectomy, 50.6% received radiotherapy, and 87% patients were treated with chemotherapy. A total of 32.5% of patients had brain metastasis, and among those who underwent surgery, 90.2% underwent lobectomy, 29.9% of patients received whole brain radiation therapy (WBRT), and 4% received prophylactic cranial irradiation (PCI), positive PD-L1 expression (Tumor Cell Proportion Score (TPS) ≥ 1%) in 18.2% (14/77) of patients, Ki-67 expression was up to 50% and above in 67.5% (52/77) of patients. The clinicopathological characteristics of the patients in the training group and the validation group are shown in Table 1.

### 3.2. Univariate and Multivariate Analyses and Screen for Predictive Factors

**Figure 1:**
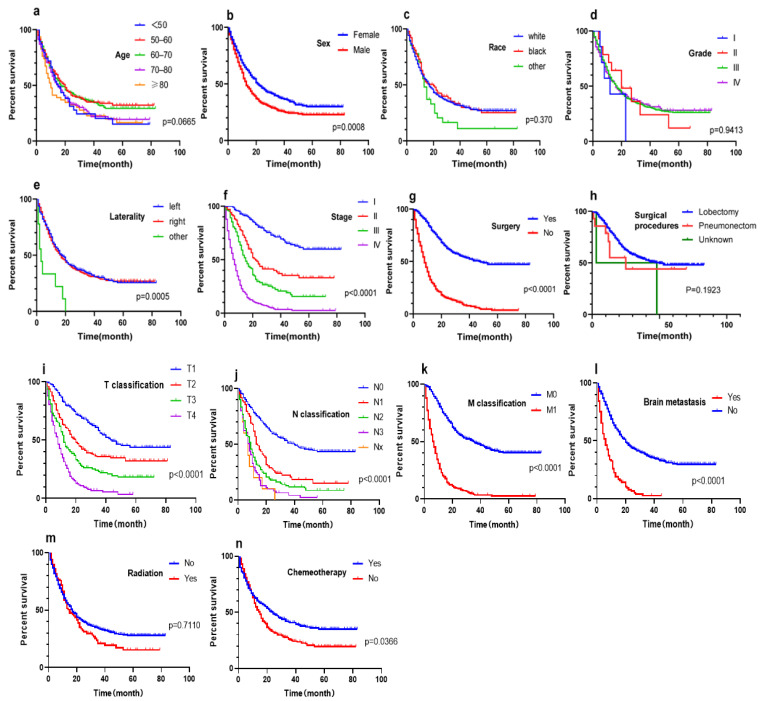
Kaplan–Meier curves of disease-specific survival (DSS) based on (**a**) age, (**b**) sex, (**c**) race, (**d**) grade, (**e**) laterality, (**f**) TMN stage, (**g**) surgery, (**h**) surgical procedure, (**i**) T classification, (**j**) N classification, (**k**) M classification, (**l**),brain metastasis, (**m**) radiation, (**n**) chemotherapy.

In the training cohort, the Cox proportional hazards model was used to assess the potential of each variable to predict DSS. Univariate analysis showed that age, gender, laterality, T classification, lymph node metastasis status, M classification, brain metastasis, TMN stage, and surgery were potential predictors of DSS in LCNEC patients (all *p* < 0.1) (Table 2). It is worth mentioning that we also used the Kaplan–Meier method to clarify the impact of relevant factors on DSS. Kaplan–Meier curves showed that age < 60 (women), negative metastasis (including brain metastasis) and distant lymph node, T1-2 classification, left laterality, early TMN stage, and acceptance of surgery and treatment were correlated with superior DSS (all *p* < 0.05) (Figure 1). We conducted a subgroup analysis of patients who underwent surgery in the training group. However, in the Kaplan–Meier survival analysis, the surgical approach was not identified as a significant predictor of DSS (*p* = 0.1923). Chemotherapy had no significance in Cox univariate analysis; however, there were differences in the Kaplan–Meier curve between the chemotherapy group and the non-chemotherapy group. It is considered that this variable may have an impact on prognosis, so it was still included in multivariate analysis. Ultimately, these above factors were included in multivariate Cox regression analysis, and the results showed that gender, T classification, N classification, M classification, surgery, and chemotherapy were independent factors affecting the prognosis of LCNEC patients (all *p* < 0.05) (Table 3).

### 3.3. Constructing and Validating the Nomogram

**Figure 2:**
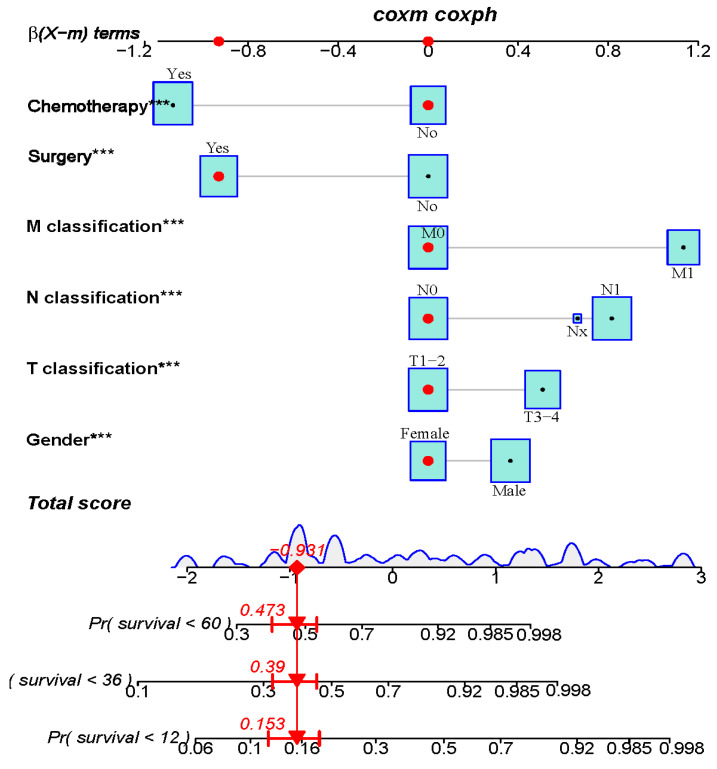
Nomogram for predicting 1, 3, and 5-year probabilities of DSS of patients with LCNEC. The model employs six predictors to generate points for each patient, which are subsequently added to obtain a total points score. The total points score is then used to determine the probability of 1-, 3-, and 5-year DSS by drawing a line down along the corresponding axis of the nomogram. The symbols “***” indicate the statistical significance level of the *p*-value of a variable. The red dots in the figure indicate the clinical information of the selected patients.

**Figure 3:**
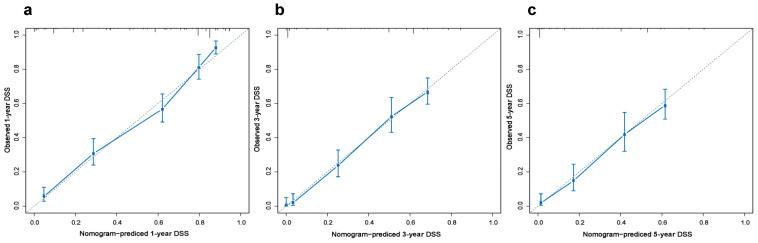
The calibration plots between the nomogram and the actual observation in the training cohort for predicting the probability of (**a**) 1-, (**b**) 3-, and (**c**) 5-year disease-specific survival (DSS). The horizontal coordinate represents the survival rate of individuals predicted by the model, and the vertical coordinate represents the actual survival of individuals.

**Figure 4:**
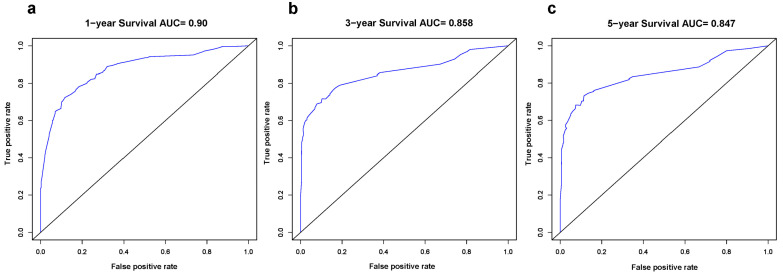
The receiver operating characteristic (ROC) curves of the nomogram for prognosis prediction at (**a**) 1, (**b**) 3, and (**c**) 5 years in the training group. It calculates a series of sensitivities and specificities by setting a number of different threshold values for continuous variables, then plots a curve with sensitivity as the vertical coordinate and (1—specificity) as the horizontal coordinate.

**Figure 5:**
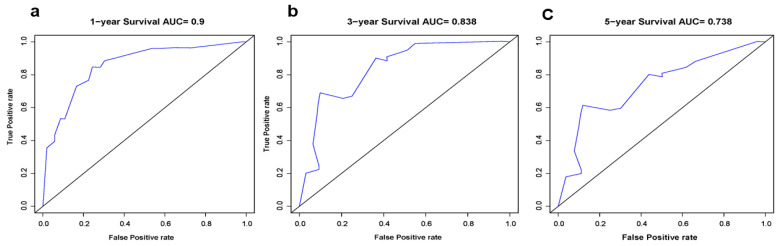
The ROC curves of the nomogram for prognosis prediction at (**a**) 1, (**b**) 3, and (**c**) 5 years in the validation group.

**Figure 6:**
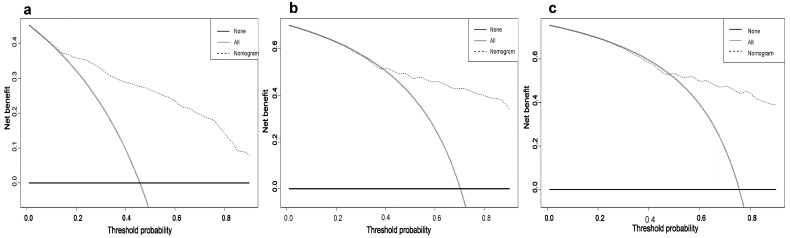
Decision curve analysis (DCA) for the Nomogram in the prediction of prognosis of LCNEC patients at (**a**) 1-, (**b**) 3-, and (**c**) 5-year points in the training cohort. The *y*-axis indicates the net benefit: and the *x*-axis indicates threshold probability. The horizontal line along the *x*-axis assumes that disease-specific death occurred in no patients, whereas the solid gray line assumes that all patients will have disease-specific death at a specific threshold probability.

Based on the results of multivariate analysis, independent predictors, such as gender, T classification, N classification, M classification, surgery, and chemotherapy were finally included in the nomogram model, and the prediction chart of DSS for LCNEC patients of 1, 3, and 5 years was obtained. Based on the patient’s medical history information, add the scores of each variable to obtain the total points. Through the total points, the corresponding 1-year, 3-year, and 5-year DSS rates on the nomogram can be acquired. The higher total scores indicated a lower corresponding survival rate (Figure 2). The C-index of this nomogram in the primary cohort and external verification cohort were 0.803 and 0.767, respectively. The calibration curve was drawn according to the individual prediction and actual survival, and a perfect prediction would be represented by a slope of 1 (a gray line with a diagonal of 45 degrees). The results showed that the 1-year, 3-year, and 5-year DSS rates predicted by the nomogram were very close to the survival curve of the actual patients in the training cohort (Figure 3). In the primary cohort, the AUC for predicting 1-, 3-, and 5-year survival was 0.90, 0.858, and 0.847, respectively. In the external validation cohort, the AUC for predicting 1-, 3-, and 5-year DSS was 0.90, 0.838, and 0.738, respectively (Figure 4 and Figure 5). These results suggest that the prognostic model exhibits relatively high sensitivity and specificity for predicting survival outcomes in patients with LCNEC, particularly for the 1-year prediction. The DCA demonstrated that the nomogram exhibited great positive net benefits among most threshold probabilities at different time points. (Figure 6). Furthermore, the C-index of this nomogram (0.803) was higher than that of the seventh edition AJCC staging system (0.755), indicating the model’s relatively accurate predictive power.

### 3.4. Risk Classification System

**Figure 7:**
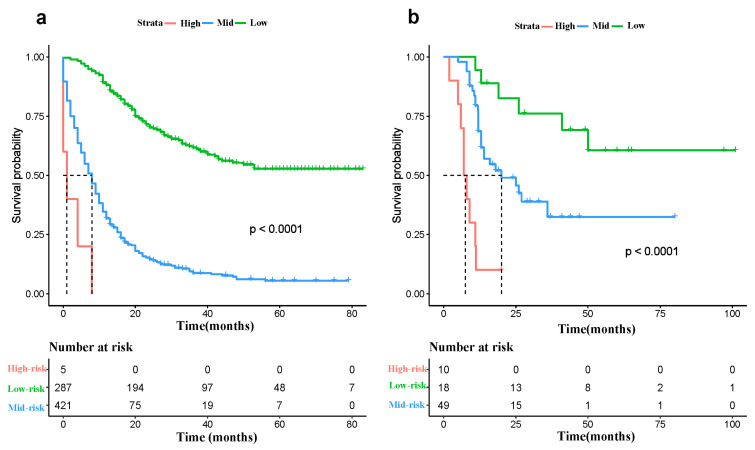
The Kaplan–Meier DSS curves for low-, medium- and high-risk patients in the training cohort (**a**) and validation cohort (**b**).

We developed a risk classification system based on the total scores generated by the nomogram for each patient. The optimal cutoff value for the risk score was calculated using X-Tiles software (Version 3.6.1), and patients were classified into low-, medium-, and high-risk groups accordingly. We then utilized Kaplan–Meier curves and Log-rank tests to determine the prognostic differences between the three groups of patients (Figure 7). The results demonstrated that the risk stratification system effectively distinguished the survival of patients in both the training and validation groups (*p* < 0.001).

### 3.5. Subgroup Analysis of West China Hospital cohort

**Figure 8:**
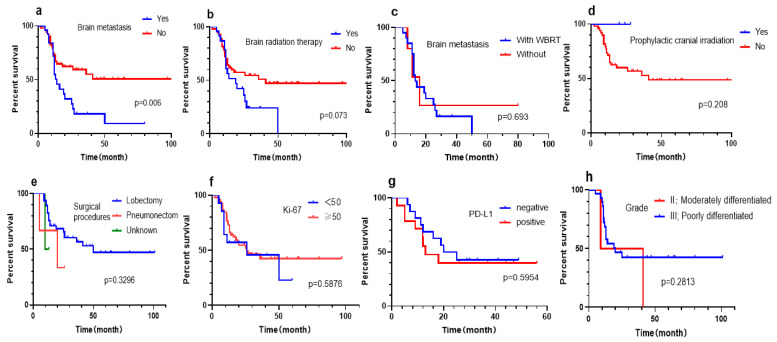
Subgroup analysis of West China hospital cohort. (**a**) brain metastasis; (**b**) brain radiation therapy; (**c**) whole brain radiation therapy (WBRT); (**d**) prophylactic cranial irradiation (PCI); (**e**) surgical procedure; (**f**) Ki-67; (**g**) PD-L1; (**h**) grade.

Some factors affecting prognosis, such as mitotic rate, Ki-67, PD-L1 expression, and the details of systemic chemotherapy and radiotherapy, were not recorded in the SEER database or with generous data missing. A total of 77 LCNEC patients from West China Hospital were enrolled in the study. As such, we conducted a subgroup analysis of available information (such as WBRT, PCI, surgical procedures, tumor grade, Ki-67, and PD-L1 expression) in external cohorts to explore the potential impact of these factors on DSS. Kaplan–Meier curve demonstrated that the prognosis of LCNEC patients with brain metastasis seemed to be significantly worse (*p* = 0.006). Additionally, brain radiation did not improve the prognosis of patients. Of the 25 patients with brain metastases, 20 received WBRT. In the same measure, WBRT was not found to be significant for brain metastases patients. The three patients who received PCI displayed a trend toward improved survival outcomes in the Kaplan–Meier curve. However, the results did not reach statistical significance. As previously mentioned, surgical procedure (*p* = 0.1923) and tumor grade (*p* = 0.9413) were not identified as significant predictors of DSS in the primary group. Similarly, we found no discernible effect of surgical approach and tumor grade on the survival of the external validation group (*p* values: 0.3296 and 0.2813, respectively). Univariate analysis was conducted to assess the effects of Ki-67 and PD-L1 expression, but neither was found to have a significant effect on survival (*p* values: 0.5876 and 0.5954, respectively) (Figure 8).

## 4. Discussion

LCNEC is an unusual, aggressive cancer with a dismal prognosis. It displays biological behaviors resembling both NSCLC and SCLC [2,6]; there are no obvious symptoms in the initial stage. Hence, approximately 60–80% of patients have lymph node metastasis, and 40% exhibit distant metastasis at the time of diagnosis [17,18]. Research indicated that LCNEC patients had a median overall survival (OS) of 10 months; once distant metastasis occurred, the average OS was only 5 months [4]. Due to the relative scarcity of cases and the lack of prospective randomized clinical trials, the prognostic characteristics of LCNEC patients have not been widely explored. At present, some studies have focused on the clinical characteristics and survival rate of LCNEC and established prediction models on this basis. However, these current prediction models were mostly based on large real-world sample data. Due to ethnic and regional differences, and a lack of independent external verification, the ability of these prognostic models to predict the risk of LCNEC in China is not clear. In this study, we obtained patients information from the SEER database to provide basic data for the analysis of prognostic factors and nomogram. In addition, to test the applicability of the nomogram to the Chinese population, LCNEC patients in West China Hospital of Sichuan University from 2010 to 2018 were selected as the external validation cohort.

Univariate and multivariate Cox regression analysis was performed by incorporating variables, such as gender, age, pathological type, treatment options, and so on; six independent prognostic factors were finally determined [9,19]. While generally similar to some previous studies, these factors were not exactly the same [19].

Previous studies have shown that gender was an independent risk factor affecting the prognosis of LCNEC patients [2]. The prognosis of male patients is poor, and it may be due to the predominance of male smokers. With a relative risk ratio of 17.40, the histological grade of neuroendocrine tumor was the most significant prognostic predictor, according to a retrospective study conducted by 10 Japanese institutes [5]. Contrary to expectations, in our research, the histological grade did not affect the patient survival; this disparity could be due to the difference in sample sizes between the two studies. The clinical stage at the time of diagnosis was also a crucial predictive factor. Derks et al. showed that the median survival (mOS) of LCNEC patients in stages I–II, III, and IV were 32.4 months (22.0–42.9 months), 12.6 months (10.3–15.0 months), and 4.0 months (3.5–4.6 months), respectively [20].

Due to a scarcity of research data, it is suggested that the surgical treatment principle of LCNEC should refer to NSCLC. Radical resection should be the first choice for operable patients (TNM stage is stage I, stage II, and partial stage IIIA) [2,21]. A large retrospective study using SEER data in patients with pulmonary LCNEC demonstrated that surgery was interrelated to improved overall survival [22], and some studies have shown that surgical treatment benefits about 30% of patients [19]. Additionally, surgery is also an important method for a definite diagnosis. The pathological manifestations of LCNEC are complex. The diagnosis obtained from smaller tissue specimens has been controversial because specific neuroendocrine patterns were difficult to see morphologically [10]. Moreover, mismatches between preoperative and postoperative pathological results were hardly new [9,12]. Therefore, surgical resection of specimens is often required to achieve a pathological diagnosis. The researchers believed that the surgical indications should be appropriately flexible for patients with suspected LCNEC. Even when metastasis is suspected, surgery should also be considered to obtain sufficient diseased tissue for a clear diagnosis [23]. In the meanwhile, due to the unique biological characteristics and high recurrence and metastasis rate, surgery alone is not enough for the treatment of LCNEC. At present, most scholars believe that adjuvant chemotherapy can improve the prognosis of LCNEC patients. Saji et al. found that patients with LCNEC who got perioperative adjuvant chemotherapy had a significantly greater survival rate than those who received surgery alone (*p* = 0.04). The 5-year survival rate for patients who had perioperative adjuvant chemotherapy was 87.5% compared to 58.5% for patients who received surgery alone. Even in stage I instances, perioperative adjuvant chemotherapy was superior to surgery alone in terms of survival. [24]. Another multicenter study also indicated that compared with no chemotherapy, preoperative or postoperative chemotherapy for stage I disease tended to improve the prognosis (*p* = 0.077) [25].Thus far, only some retrospective studies and a small prospective study have shown that patients with LCNEC who received a chemotherapy regimen for SCLC, namely, Etoposide + Cisplatin (EP) or Etoposide + carboplatin (EC) regimen, had a higher survival rate [13,26]. Nevertheless, due to SEER database restrictions, it was not possible to collect information about the details of chemotherapy regimens, which impeded further prognostic analysis based on detailed chemotherapy regimens. Up to now, the clinical benefit of radiation for LCNEC is controversial, and some scholars believed that radiotherapy can be tried for patients with limited lesions, progressive stage, or inappropriate for surgery [27]. Jiang et al.conducted a retrospective analysis to evaluate the effect of surgery and radiotherapy on patients with LCNEC; they found that radiotherapy may reduce survival time in patients undergoing surgery. However, for patients with stage III LCNEC, radiotherapy may have a positive impact on survival time, especially for patients who are not suitable for surgical resection [28]. Another retrospective study of SEER data revealed that postoperative radiotherapy could not improve the long-term prognosis of LCNEC patients. There was no significant difference in survival between patients with or without postoperative radiotherapy (*p* = 0.489), even when subgroups were deeply analyzed [29]. In our study, radiation did not show a significant effect on DSS, and the Kaplan–Meier curve demonstrated that patients with radiotherapy had worse DSS. This difference may be due to the fact that postoperative adjuvant radiotherapy is mainly concentrated on locally advanced and recurrent lesions, but the survival rate of patients with these lesions is low owing to the late stage of the tumor. Further subgroup analysis showed that WBRT did not improve the prognosis of patients. However, PCI appeared to have survival benefits in LCNEC patients without brain metastases, although the difference is not statistically significant. The effect of PCI on LCNEC has been controversial. Rieber et al. considered that PCI in patients with LCNEC should be thoroughly reconsidered, especially in the early stage of the tumor. They pointed out that only a few (25%) patients with LCNEC had brain metastasis, and there was a strong correlation between pathological stage and brain metastasis [30]. In retrospective research, LCNEC patients treated with PCI had longer median progression-free survival (20.5 vs. 6.4 months) and median overall survival (33.4 vs. 8.6 months) [31], though it did not reach statistical significance, such as our research. This may be due to the limited number of cases. Ki-67 is a nuclear antigen that serves as a reliable marker for assessing the proliferation status of cells. Previous studies have suggested that Ki-67 might be a promising prognostic factor in low- to intermediate-grade lung neuroendocrine (NE) tumors. However, the results are sometimes conflicting and inconclusive, making it difficult to endorse the role of Ki-67 as a prognostic factor in lung NE tumors [32,33]. In our subgroup analysis, Ki-67 expression is not an independent predictor of DSS. However, given the limited sample size, a larger-scale analysis is necessary to confirm these findings. Currently, there are few studies on the expression of PD-L1 in LCNEC. A retrospective study initiated by Eichhorn et al. revealed that positive PD-L1 expression in LCNEC was associated with poorer survival [34], which is consistent with the trend of our KM survival curve (median survival in PD-L1 positive and negative patients: 13 vs. 22.5 months). However, the results were not statistically significant (*p* > 0.05).

There were a few existing studies in which a nomogram was constructed to predict the prognosis of LCNEC patients [35,36]. However, the endpoint of most of these studies was OS. Only one study was conducted in the setting of DSS. However, this study was based on the SEER database, lacking Asian population data for validation [16]. In our research, this nomogram was evaluated by external validation with Chinese cohorts. The results of external verification proved that the predictive nomogram from SEER can also be available to the Chinese population. In addition, based on the total score predicted by the nomogram, the total population was divided into three risk stratification to verify the practicability of this prediction model. Additionally, the DCA also demonstrated the favorable clinical applicability of the prediction model.

This research still had some limitations. First, constrained by the retrospective data analysis and nonrandomization, there was inevitable internal bias and limited signification. Second, due to the limited variables collected by the SEER database, detailed information on treatment options, tumor markers, molecular typing, and other important indicators is not available, which hinders further prognostic analysis. The approach to radiotherapy and chemotherapy may have been heterogeneous between the SEER and validation sets, which may have influenced our results. Due to the low incidence of LCNEC, the number of patients that can be recruited in the validation group is small, which limits the statistical power of subgroup analyses. Therefore, in the future, we will attempt to integrate and analyze LCNEC patient data from multiple centers in China and foreign databases. Additionally, extending the follow-up time can improve the predictive value of the model.

## 5. Conclusions

The study demonstrated that the newly developed nomogram had promising prognostic potential and clinical applicability in predicting outcomes of patients diagnosed with LCNEC. However, it is imperative to expand the sample size in future investigations and extend the follow-up duration to enhance the precision of the prognostic model.

## Figures and Tables

**Table 1:** Clinicopathological features and treatment background of all training and validation cohort patients at baseline.

Characteristics	Training Cohort N = 713	Validation Cohort N = 77	*p* Value *
Age			0.0007
<50	40 (5.6%)	5 (6.4%)	
50–59	167 (23.4%)	32 (41.6%)	
60–69	276 (38.7%)	31 (40.3%)	
70–79	177 (24.8%)	8 (10.4%)	
≥80	53 (7.5%)	1 (1.3%)	
Sex			<0.0001
Female	323 (45.3%)	9 (11.7%)	
Male	390 (54.7%)	68 (88.3%)	
Race			<0.0001
White	589 (82.6%)	0	
Black	95 (13.3%)	0	
Other	29 (4.1%)	77 (100%)	
Laterality			0.4721
Left	291 (40.8%)	37 (48.1%)	
Right	411 (57.6%)	39 (50.6%)	
Other (Bilateral and Paired site)	11 (1.6%)	1 (1.3%)	
T classification			0.8490
T1-2	397 (55.7%)	42 (54.5%)	
T3-4	316 (44.3%)	35 (45.5%)	
N classification			0.0054
N0	342 (48%)	24 (31.2%)	
N1-3	357 (50%)	53 (68.8%)	
Nx	14 (2%)	0	
M classification			0.2213
M0	429 (60.2%)	52 (67.5%)	
M1	284 (39.8%)	25 (32.5%)	
AJCC Stage			0.0004
I	193 (27.1%)	9 (11.7%)	
II	100 (14%)	16 (20.8%)	
III	136 (19.1%)	27 (35.1%)	
IV	284 (39.8%)	25 (32.4%)	
Brain metastasis			<0.0001
Yes	99 (13.9%)	25 (32.5%)	
No/Unknown	614 (86.1%)	52 (67.5%)	
Surgery			0.0026
Yes	342 (48%)	51 (66.2%)	
No/unknown	371 (52%)	26 (33.8%)	
Radiation			<0.0001
Yes	102 (14.3%)	39 (50.6%)	
No/unknown	611 (85.7%)	38 (49.4%)	
Chemotherapy			<0.0001
Yes	396 (55.5%)	67 (87%)	
No/unknown	317 (44.5%)	10 (13%)	
Surgical procedures			0.1595
Lobectomy	325 (95.0%)	46 (90.2%)	
Pneumonectomy	14 (4.1%)	3 (5.9%)	
Unknown	3 (0.9%)	2 (3.9%)	
Grade		6 (7.8%) 38 (49.4%)	/
Grade I	7 (1%)		
Grade II	14 (2%)		
Grade III	522 (73.2%)		
Undifferentiated	170 (23.8%)		
Ki-67			/
<50%	/	14 (18.2%)	
≥50%	/	52 (67.5%)	
Unknown	/	11 (14.3%)	
PD-L1			/
Negative (TPS < 1%)	/	16 (20.8%)	
Positive	/	14 (18.2%)	
Unknown	/	47 (61%)	
Whole brain radiotherapy			
Yes	/	23 (29.9%)	/
No	/	54 (70.1%)	
Prophylactic-cranial irradiation			
Yes	/	3 (4%)	
No	/	74 (96%)	/

* *p* > 0.05.

**Table 2:** Univariate analysis of the capacity of each factor to predict DSS.

Univariate Analyses	HR	95% CI	*p* Value †	C-Index
Age (vs. <50)			0.011	0.546
50–59	0.767	0.514–1.145		
60–69	0.837	0.572–1.225		
70–79	1.077	0.728–1.594		
≥80	1.266	0.799–2.006		
Sex			<0.001	0.551
Male vs. Female	1.412	1.180–1.690		
Race			0.281	0.51
Black vs. White	0.949	0.732–1.231		
Other vs. White	1.363	0.910–2.042		
Grade			0.959	0.501
II vs. I	0.751	0.257–2.199		
III vs. I	0.863	0.357–2.087		
IV vs. I	0.856	0.349–2.098		
Laterality			0.001	0.519
Right vs. left	1.051	0.877–1.261		
Other vs. left	3.335	1.813–6.137		
T classification			<0.001	0.67
T3-4 vs. T1-2	2.684	2.240–3.215		
N classification			<0.001	0.676
N1-3 vs. N0	3.217	2.656–3.896		
Nx vs. N0	6.196	3.575–10.740		
M classification			<0.001	0.698
M1 vs. M0	4.991	4.138–6.021		
Brain metastasis			<0.001	0.569
Yes vs. No	3.098	2.462–3.898		
AJCC stage			<0.001	0.755
II vs. I	2.57	1.790–3.689		
III vs. I	4.426	3.199–6.123		
IV vs. I	10.928	8.151–14.653		
Radiation			0.4	0.497
Yes vs. No	1.11	0.871–1.413		
Surgery			<0.001	0.691
Yes vs. No	0.218	0.170–0.265		
Chemotherapy			0.284	0.483
Yes vs. No	1.103	0.922–1.321		

† *p* < 0.05. Abbreviations: DSS, disease-specific survival; HR, hazard ratio; CI, confidence interval; C-index, the concordance index.

**Table 3:** Multivariate analysis of the capacity of each factor to predict DSS.

Multivariate Analysis	HR	95% CI	*p* Value ^‡^
Sex			
Male vs. Female	1.529	1.273–1.837	<0.001
T classification			
T3-4 vs. T1-2	1.676	1.368–2.054	<0.001
N classification			
N1-3 vs. N0	2.324	1.858–2.906	<0.001
Nx vs. N0	1.953	1.092–3.491	0.024
M classification			
M1 vs. M0	3.292	2.620–4.137	<0.001
Surgery			
Yes vs. No	0.415	0.321–0.537	<0.001
Chemotherapy			
Yes vs. No	0.356	0.288–0.440	<0.001

^‡^ *p* < 0.05. Abbreviations: DSS, disease-specific survival; HR, hazard ratio; CI, confidence interval.

## Data Availability

These data were derived from the following resources available in the public domain: the US National Cancer Institute’s Surveillance Epidemiology and End Results (SEER) registry.

## References

[1] Hendifar A.E., Marchevsky A.M., Tuli R. (2017). Neuroendocrine Tumors of the Lung: Current Challenges and Advances in the Diagnosis and Management of Well-Differentiated Disease. J. Thorac. Oncol..

[2] Gollard R., Jhatakia S., Elliott M., Kosty M. (2010). Large cell/neuroendocrine carcinoma. Lung Cancer.

[3] Travis W.D., Linnoila R.I., Tsokos M.G., Hitchcock C.L., Cutler G.B., Nieman L., Chrousos G., Pass H., Doppman J. (1991). Neuroendocrine tumors of the lung with proposed criteria for large-cell neuroendocrine carcinoma. An ultrastructural, immunohistochemical, and flow cytometric study of 35 cases. Am. J. Surg. Pathol..

[4] Shah S., Gosain R., Groman A., Gosain R., Dasari A., Halfdanarson T.R., Mukherjee S. (2021). Incidence and Survival Outcomes in Patients with Lung Neuroendocrine Neoplasms in the United States. Cancers.

[5] Asamura H., Kameya T., Matsuno Y., Noguchi M., Tada H., Ishikawa Y., Yokose T., Jiang S.X., Inoue T., Nakagawa K. (2006). Neuroendocrine neoplasms of the lung: A prognostic spectrum. J. Clin. Oncol..

[6] Iyoda A., Makino T., Koezuka S., Otsuka H., Hata Y. (2014). Treatment options for patients with large cell neuroendocrine carcinoma of the lung. Gen. Thorac. Cardiovasc. Surg..

[7] Lo Russo G., Pusceddu S., Proto C., Macerelli M., Signorelli D., Vitali M., Ganzinelli M., Gallucci R., Zilembo N., Platania M. (2016). Treatment of lung large cell neuroendocrine carcinoma. Tumour Biol..

[8] Chen Y., Zhang J., Huang C., Tian Z., Zhou X., Guo C., Liu H., Li S. (2021). Survival outcomes of surgery in patients with pulmonary large-cell neuroendocrine carcinoma: A retrospective single-institution analysis and literature review. Orphanet J. Rare Dis..

[9] Eichhorn F., Dienemann H., Muley T., Warth A., Hoffmann H. (2015). Predictors of survival after operation among patients with large cell neuroendocrine carcinoma of the lung. Ann. Thorac. Surg..

[10] Hiroshima K., Abe S., Ebihara Y., Ogura S., Kikui M., Kodama T., Komatsu H., Saito Y., Sagawa M., Sato M. (2005). Cytological characteristics of pulmonary large cell neuroendocrine carcinoma. Lung Cancer.

[11] Garcia-Yuste M., Matilla J.M., Alvarez-Gago T., Duque J.L., Heras F., Cerezal L.J., Ramos G. (2000). Prognostic factors in neuroendocrine lung tumors: A Spanish Multicenter Study. Spanish Multicenter Study of Neuroendocrine Tumors of the Lung of the Spanish Society of Pneumonology and Thoracic Surgery (EMETNE-SEPAR). Ann. Thorac. Surg..

[12] Takei H., Asamura H., Maeshima A., Suzuki K., Kondo H., Niki T., Yamada T., Tsuchiya R., Matsuno Y. (2002). Large cell neuroendocrine carcinoma of the lung: A clinicopathologic study of eighty-seven cases. J. Thorac. Cardiovasc. Surg..

[13] Rossi G., Cavazza A., Marchioni A., Longo L., Migaldi M., Sartori G., Bigiani N., Schirosi L., Casali C., Morandi U. (2005). Role of chemotherapy and the receptor tyrosine kinases KIT, PDGFRalpha, PDGFRbeta, and Met in large-cell neuroendocrine carcinoma of the lung. J. Clin. Oncol..

[14] Liang W., Zhang L., Jiang G., Wang Q., Liu L., Liu D., Wang Z., Zhu Z., Deng Q., Xiong X. (2015). Development and validation of a nomogram for predicting survival in patients with resected non-small-cell lung cancer. J. Clin. Oncol..

[15] Iasonos A., Schrag D., Raj G.V., Panageas K.S. (2008). How to build and interpret a nomogram for cancer prognosis. J. Clin. Oncol..

[16] Ma H., Xu Z., Zhou R., Liu Y., Zhu Y., Chang X., Chen Y., Zhang H. (2021). A Clinical Nomogram for Predicting Cancer-Specific Survival in Pulmonary Large-Cell Neuroendocrine Carcinoma Patients: A Population-Based Study. Int. J. Gen. Med..

[17] Sanchez de Cos Escuin J. (2014). Diagnosis and treatment of neuroendocrine lung tumors. Arch. Bronconeumol..

[18] Foster N.R., Qi Y., Shi Q., Krook J.E., Kugler J.W., Jett J.R., Molina J.R., Schild S.E., Adjei A.A., Mandrekar S.J. (2011). Tumor response and progression-free survival as potential surrogate endpoints for overall survival in extensive stage small-cell lung cancer: Findings on the basis of North Central Cancer Treatment Group trials. Cancer.

[19] Fournel L., Falcoz P.E., Alifano M., Charpentier M.C., Boudaya M.S., Magdeleinat P., Damotte D., Regnard J.F. (2013). Surgical management of pulmonary large cell neuroendocrine carcinomas: A 10-year experience. Eur. J. Cardiothorac. Surg..

[20] Derks J.L., Hendriks L.E., Buikhuisen W.A., Groen H.J., Thunnissen E., van Suylen R.J., Houben R., Damhuis R.A., Speel E.J., Dingemans A.M. (2016). Clinical features of large cell neuroendocrine carcinoma: A population-based overview. Eur. Respir. J..

[21] Doddoli C., Barlesi F., Chetaille B., Garbe L., Thomas P., Giudicelli R., Fuentes P. (2004). Large cell neuroendocrine carcinoma of the lung: An aggressive disease potentially treatable with surgery. Ann. Thorac. Surg..

[22] Kinslow C.J., May M.S., Saqi A., Shu C.A., Chaudhary K.R., Wang T.J.C., Cheng S.K. (2020). Large-Cell Neuroendocrine Carcinoma of the Lung: A Population-Based Study. Clin. Lung Cancer.

[23] Welter S., Aigner C., Roesel C. (2017). The role of surgery in high grade neuroendocrine tumours of the lung. J. Thorac. Dis..

[24] Saji H., Tsuboi M., Matsubayashi J., Miyajima K., Shimada Y., Imai K., Kato Y., Usuda J., Kajiwara N., Uchida O. (2010). Clinical response of large cell neuroendocrine carcinoma of the lung to perioperative adjuvant chemotherapy. Anticancer Drugs.

[25] Veronesi G., Morandi U., Alloisio M., Terzi A., Cardillo G., Filosso P., Rea F., Facciolo F., Pelosi G., Gandini S. (2006). Large cell neuroendocrine carcinoma of the lung: A retrospective analysis of 144 surgical cases. Lung Cancer.

[26] Filosso P.L., Guerrera F., Evangelista A., Galassi C., Welter S., Rendina E.A., Travis W., Lim E., Sarkaria I., Thomas P.A. (2017). Adjuvant chemotherapy for large-cell neuroendocrine lung carcinoma: Results from the European Society for Thoracic Surgeons Lung Neuroendocrine Tumours Retrospective Database. Eur. J. Cardiothorac. Surg..

[27] Sarkaria I.S., Iyoda A., Roh M.S., Sica G., Kuk D., Sima C.S., Pietanza M.C., Park B.J., Travis W.D., Rusch V.W. (2011). Neoadjuvant and adjuvant chemotherapy in resected pulmonary large cell neuroendocrine carcinomas: A single institution experience. Ann. Thorac. Surg..

[28] Jiang H., Wu Q., Zhong Y. (2021). Survival and prognosis of lung large cell neuroendocrine carcinoma. Bull. Cancer.

[29] Gang J., Lili X., Xiang S. (2020). The role of postoperative radiotherapy (PORT) in pulmonary large cell neuroendocrine carcinoma (PLCNEC). Cancer Radiother..

[30] Rieber J., Schmitt J., Warth A., Muley T., Kappes J., Eichhorn F., Hoffmann H., Heussel C.P., Welzel T., Debus J. (2015). Outcome and prognostic factors of multimodal therapy for pulmonary large-cell neuroendocrine carcinomas. Eur. J. Med. Res..

[31] Prelaj A., Rebuzzi S.E., Del Bene G., Giron Berrios J.R., Emiliani A., De Filippis L., Prete A.A., Pecorari S., Manna G., Ferrara C. (2017). Evaluation of the efficacy of cisplatin-etoposide and the role of thoracic radiotherapy and prophylactic cranial irradiation in LCNEC. ERJ Open Res..

[32] Igarashi T., Jiang S.X., Kameya T., Asamura H., Sato Y., Nagai K., Okayasu I. (2004). Divergent cyclin B1 expression and Rb/p16/cyclin D1 pathway aberrations among pulmonary neuroendocrine tumors. Mod. Pathol..

[33] Pelosi G., Rindi G., Travis W.D., Papotti M. (2014). Ki-67 antigen in lung neuroendocrine tumors: Unraveling a role in clinical practice. J. Thorac. Oncol..

[34] Eichhorn F., Harms A., Warth A., Muley T., Winter H., Eichhorn M.E. (2018). PD-L1 expression in large cell neuroendocrine carcinoma of the lung. Lung Cancer.

[35] He Y., Liu H., Wang S., Chen Y. (2019). Prognostic nomogram predicts overall survival in pulmonary large cell neuroendocrine carcinoma. PLoS ONE.

[36] Xi J., Zhao M., Zheng Y., Liang J., Hu Z., Huang Y., Yang Y., Zhan C., Jiang W., Lu T. (2020). Development and validation of a nomogram for predicting the overall survival of patients with lung large cell neuroendocrine carcinoma. Transl. Cancer Res..

